# Treatment-related toxicity, utility and patient-reported outcomes of head and neck cancer patients treated with proton therapy: A longitudinal study

**DOI:** 10.1016/j.ctro.2025.100913

**Published:** 2025-01-09

**Authors:** Yi Hsuan Chen, Michiel Kroesen, Mischa Hoogeman, Matthijs Versteegh, Carin Uyl-de Groot, Hedwig M. Blommestein

**Affiliations:** a Erasmus School of Health Policy & Management Erasmus University Rotterdam Rotterdam the Netherlands; b Department of Medical Physics and Informatics HollandPTC Delft the Netherlands; c Department of Radiotherapy Erasmus MC Cancer Institute University Medical Center Rotterdam Rotterdam the Netherlands; d Institute for Medical Technology Assessment, Erasmus University Rotterdam, Burgemeester Oudlaan 50 3062 PA Rotterdam, the Netherlands

**Keywords:** Proton therapy, Head and neck cancer, Quality of life, Health-related quality of life, Radiotherapy-related toxicity

## Abstract

**Objective:**

In comparison to current standard photon irradiation, proton therapy (PT) significantly reduces dose to the surrounding normal tissue and therefore is expected to reduce toxicity and improve health related quality of life (HRQoL). Despite the high expectations of PT, there is very limited data on patients’ HRQoL after radiotherapy. This study evaluated HRQoL in head and neck cancer (HNC) patients receiving PT and established a robust benchmark for future comparison of PT and the radiotherapy advancements.

**Method:**

A questionnaire-based (consisting of EORTC-QLQ-C30, EQ-5D, and EORTC-H&N-35) prospective cohort study was performed in a Dutch proton therapy center. HNC patients who received PT between January 2020 to December 2022 were enrolled in this study. The questionnaires were distributed pre-treatment, and 0, 6, 12, 24 months post-treatment. The generalized estimating equations method was used to analyze the utility change and negative impact of the radiation-related toxicities.

**Results:**

119 HNC patients were included in the study. Symptom and function scores showed the deterioration of all reported functions during the period of treatment. Most of the functions recovered within six months and improved beyond baseline. At the end of PT, the patients’ utility decreased significantly (0.12 points) compared to the baseline. The loss in utility was recovered after six months and a further improvement was seen one year after the treatment. This study further provided the estimation of the disutility of each radiation related toxicity.

**Conclusion:**

The present study presented the impact of toxicity on patient’s utility over time and further confirmed it with the results of patient-reported symptom and function. This study provided estimation of each radiation-related toxicity, including xerostomia, dysphagia, mucositis, and dermatitis, which could contribute to the value assessment through economic evaluations of PT.

## Highlights

• We present a comprehensive analysis of head and neck cancer patients' utility and symptom changes after proton therapy.
• This study observed a drop in patients' utility at treatment's end, with substantial recovery within six months.
• We reported the six-month recovery period for the majority of treatment-related symptoms and functional impairments.
• Our observations highlight the significant impact of inter-toxicity correlations on treatment-related disutility.
• We advocate researchers engaged in the assessment of radiotherapy account for the inter-correlations among toxicities.

## Introduction

Head and neck cancer (HNC) is one of the most prevalent cancers worldwide, with an annual incidence of 1.1 million new cases, 4.1 million prevalent cases and causing more than 500,000 deaths in 2016 [1]. With the contribution of radiotherapy, the prognosis of HNC has been improved considerably [2]. Nowadays, radiotherapy is involved in most treatments for HNC [3]. Despite the undeniable treatment benefit of radiotherapy, HNC patients following radiotherapy suffer from radiation-related toxicities. To lower the risk of toxicities, radio oncologists and physicians have focused on developing new treatment approaches and adopting the innovations.

Proton Therapy (PT) could better preserve normal tissues surrounding the tumor. This benefit comes from the finite range of protons, as opposed to conventionally used photons that deposit dose along their entire path through the patient. Due to this advantage, PT has been reported to have a lower rate of acute radiation-related toxicities [4], a lower rate of gastrostomy tube use [5], and less need for pain control [6] compared with photon radiotherapy.

The advantages imply the potential of using HNC as a suitable indication for PT. Nevertheless, to make a holistic judgment of its treatment effects, the estimation and validation of the health-related quality of life (HRQoL) in HNC patients is imperative. This information is crucial for understanding the subjective experiences, the perceived clinical outcomes by patients, and assessing the burdens of symptoms from patients’ perceptions. Patient-reported outcomes could contribute to enhancing patient-centered care, fostering shared decision-making, and ultimately improving healthcare quality. Additionally, a well-established set of patient-reported outcomes could serve as a fundament for facilitating real-time comparisons of future innovative approaches with PT.

A comprehensive analysis of HRQoL requires both disease-specific questionnaires and generic instruments. Disease-specific questionnaires enable a thorough examination of changes in disease-related functions and symptoms over time. Conversely, utility metrics capture the preferences of the general population regarding living with specific symptoms. Derived from patient questionnaires and population-preference tariffs, utility metrics yield quantified insights exceeding specific diseases, rendering them valuable instruments for policymakers.

While there are several studies available that investigated the impact of HNC treatment in general [7], [8], [9], [10], existing evidence on the impact of toxicity on patients' HRQoL or utility is sparse. Only two studies explicitly described the negative influence of radiation-related toxicity on patients' HRQoL or utility in patients who received photon radiotherapy [11], [12]. Notably, both studies described only the negative influence of xerostomia and dysphagia. Moreover, due to the different instruments used, the results of these two studies are not suitable to be directly compared. While Sharma et al. measured HRQoL post-PT, utility instruments were not included and therefore changes in post-PT in HNC patients are currently unavailable [13]. This underscores the urgent need for further exploration and analysis in this domain to describe the overall health related quality of life of patients receiving PT and the impact of toxicities.

This study aims to provide a cornerstone for the HRQoL of an early cohort of patients with HNC who have undergone PT in the Netherlands. To achieve this, the research adopted a dual approach, integrating disease-specific and generic quality of life questionnaires. Furthermore, the study sought to quantify the impact of radiation-induced toxicities on patients' quality of life, accounting for multiple factors such as patient demographics, cancer characteristics, and types of treatment. Quantifying the impact of radiation-induced toxicities is crucial to pave the way for assessing the value and cost-effectiveness of future radiotherapy advancements.

## Method

### Study population

A questionnaire-based prospective cohort study was performed at the Holland Proton Therapy Center (HollandPTC). In the Netherlands, patients are selected according to a national indication protocol that involves a normal tissue complication probability (NTCP) comparison based on a PT and a radiotherapy treatment plan. If the NTCP reduction by PT exceeds a predefined threshold, depending on the toxicity grade, the patient can be considered eligible for PT [14]. All HNC patients who received PT at HollandPTC, including definitive, postoperatively, and re-irradiation, between January 2020 to December 2022 were invited to enroll in this study. All HNC patients were treated with a dose of 70 GyRBE to gross disease areas (Clinical Target Volume 7000, CTV7000) and 54.25 GyRBE to elective regions (CTV5425, which includes CTV7000), based on a constant Relative Biological Effectiveness (RBE) of 1.1. The treatment was administered in 35 fractions, with target coverage constraints set to ensure that at least 98 % of the prescribed dose was reached in the minimum dose distribution for both CTVs. Patients enrolled in this study all provided written informed consent. This study was approved by the ethics committee (METC Leiden University Medical Center (reference number P18/053/SH/sh)).

### Data collection

The questionnaires were given to the patient in person during the scheduled visits. The questionnaires were distributed at the following five-time points: before the start of PT, at the end of PT, six months, one year, and two years after the PT. The eligible patients were followed up for two years after the end of PT.

Both general (i.e., EQ-5D-5L) and disease-specific questionnaires (i.e., EORTC QLQ-C30 and EORTC QLQ-H&N35) were used to measure patients' quality of life. The EQ-5D-5L questionnaire contains five dimensions: mobility, self-care, daily activities, pain/discomfort, and anxiety/depression, along with a visual analog scale (VAS) [15]. Each dimension ranged from one to five, whereas one stands for no problem and five indicates extreme problems. VAS is a single score ranging from zero to a hundred, which zero meaning the worst health condition and a hundred for the best health condition the patient can imagine.

The EORTC QLQ-C30 questionnaire (version 3.0) collects five function scales, nine disease-related symptom scales, and a global health status scale [16]. Each question's answer ranged from one to four, which betoken “Not at All”, “A Little”, “Quite a bit”, and “Very Much”, respectively.

The other disease-specific questionnaire used is EORTC QLQ-H&N35, which comprises eighteen HNC-specific symptoms [17]. Seven multi-item symptom scales (i.e., pain in the mouth, swallowing, senses, social eating, social contact, speech and sexuality), six single-item symptom scale (i.e., mouth opening, dry mouth, teeth, coughing, feeling ill, sticky saliva), and five yes or no questions (i.e., use of pain-control medicine, feeding tube, nutritional supplements, weight gain and weight loss) were included in this questionnaire. The patient's demographic and clinical data (e.g., gender, age, tumor site, smoking, and alcohol use) were obtained from the electronic medical record system of HollandPTC.

Furthermore, radiation-related toxicities were recorded and graded by radiation oncologists. The grading ranged from one to five based on the Common Terminology Criteria for Adverse Events (CTCAEv5.0) [18]. The patient’s HPV status is tested by the result of standard p16 test.

### Statistical analysis

Missing data in patients’ tumor staging (TNM), Karnofsky performance score (KPS), and chemotherapy were replaced by multiple imputations (MI) (missing percentage showed in Table 1). In contrast, the missing data of smoking and alcohol use were not imputed and remained as unknown. STATA statistical software was used for the multiple imputation procedure. Ten imputed datasets were created. A comparison between complete case analysis and MI analysis was conducted.

**Table 1:** Patient characteristics and demographic (n = 119).

**Variable**	**Number; Mean (Range; %)**
Gender	
Male	68.6 %
Age	61.5 (12.8; 20–86)
Smoking	
Never	14 (11.8 %)
Stopped	30 (25.2 %)
Yes	16 (13.4 %)
Unknown	59 (49.6 %)
Alcohol	
No	51 (42.8 %)
Stopped	8 (6.7 %)
Yes	36 (30.3 %)
Unknown	24 (20.2 %)
Tumor site	
Oral	11 (9.2 %)
Oropharynx	59 (49.6 %)
Hypopharynx	6 (5.0 %)
Nasopharynx	11 (9.2 %)
Nasal cavity	3 (2.5 %)
Unknown Primary	29 (24.4 %)
T stage	
0	4 (3.4 %)
1	22 (18.5 %)
2	50 (42.0 %)
3	19 (16.0 %)
4	24 (20.2 %)
N stage	
0	22 (18.5 %)
1	46 (38.6 %)
2	46 (38.6 %)
3	5 (4.2 %)
TNM Staging	
2	9 (7.6 %)
3	46 (38.7 %)
4	64 (53.8 %)
Chemotherapy	
No	42 (35.3 %)
Yes	77 (64.7 %)
Surgery	10 (8.3 %)
HPV (P16)*	
Negative	31 (26.1 %)
Positive	48 (40.3 %)
Test not performed	40 (33.6 %)
Karnofsky performance status at baseline	
70	2 (1.7 %)
80	11 (9.2 %)
90	61 (51.3 %)
100	24 (20.2 %)
Unknown	21 (17.6 %)
Disease progression	
Local recurrence	6 (5 %)
Regional recurrence	4 (3.3 %)
Metastasis	3 (2.5 %)
Mean dose on OAR for pharynx tumor (GyE) of PT/IMRT	
Parotid contralateral	18.4/22.8
Parotid ipsilateral	27.6/30.6
Submandibular glands	48.6/52.4
Oral cavity	26.6/40.8
Superior PCM	52.7/58.2
Medius PCM	38.3/46.1
Inferior PCM	34.9/41.8
Mean dose on OAR for larynx tumor (GyE) of PT/IMRT	
Parotid contralateral	11.5/14.6
Parotid ipsilateral	36.6/37.1
Submandibular glands	31.9/36.1
Oral cavity	11.4/24.7
Superior PCM	31.3/40.6
Medius PCM	24.5/29.7
Inferior PCM	35.4/39.9
Mean dose on OAR for oral cavity tumor (GyE) of PT/IMRT	
Parotid contralateral	7.9/10.6
Parotid ipsilateral	31.7/28.4
Submandibular glands	28.6/32.4
Oral cavity	25.0/36.5
Superior PCM	26.6/36.2
Medius PCM	18.7/28.4
Inferior PCM	18.5/27.3

Abbreviation: OAR: organ at risk; PCM: pharyngeal constrictor muscle*: The HPV status is indicated by the standard p16 protein test of cancer cells.

The utility was calculated with the EQ-5D-5L results using the Dutch value set with a utility range from −0.446 to 1 [19]. A score of one in utility indicates the perfect health condition, and zero indicates death. The negative score expresses that the health state is considered worse than death.

The scores for each domain of QLQ-C30 and QLQ-H&N35 were calculated according to the EORTC scoring procedures as outline in EORTC scoring manual [20]. Each domain was calculated from the average score of a cluster of questions (Equation 1–3) and converted to scales ranging from zero to a hundred. A higher score on functional scales suggests a better functional condition, while a higher score on symptom scales implies a weightier burden.

(1) $$\mathrm{Rawscore}=\left(I_{1}+I_{2}+\cdots +I_{n}\right)/n$$

(2) $$\mathrm{Functionalscales}=\left\{1-\frac{\left(RawScore-1\right)}{\mathrm{Range}}\right\}\times 100$$

(3) $$\mathrm{Symptomscales}=\left\{\frac{\left(RawScore-1\right)}{\mathrm{Range}}\right\}\times 100$$

*I: the score of each question

*n: number of questions in the domain

The summary C30 score was calculated averaging across all domains excluding the global health status and the financial problems (Equation 4).

(4) *Summary C30 score* = [*Physical Function + Role Function + Social Function + Emotional Function + Cognitive Function +* (*100-Fatigue*) *+* (*100-Pain*) *+* (*100-Nausea) +* (*100-Dyspnoea*) *+*(*100- Insomnia*) *+* (*100-Appetite Loss*) *+* (*100-Constipation*) *+* (*100-Diarrhoea)*]*/13*

Since our data had repeated measures and non-normal response variables, the generalized estimating equation (GEE) population average model was used. The GEE model is a method for analyzing correlated data, that can account for within-subject or within-cluster correlation and estimate the effect of covariates on the outcome variable. This model was employed in estimating the changes in utility, VAS, summary score, patient’s function, and burden of symptoms over time with controlling of patients' cancer site and staging, human papillomavirus (HPV) status, and disease progression. The disutility of radiation-related toxicities was further estimated from the GEE model on patients’ utilities controlling with patients’ disease progression. The selection of the variables for each model is based on their association to the quality-of-life outcomes. All statistical analyses were performed using STATA (Stata Statistical Software: Release 17. College Station, TX: StataCorp LLC.).

## Result

### Patients and descriptive results

**Fig. 1:**
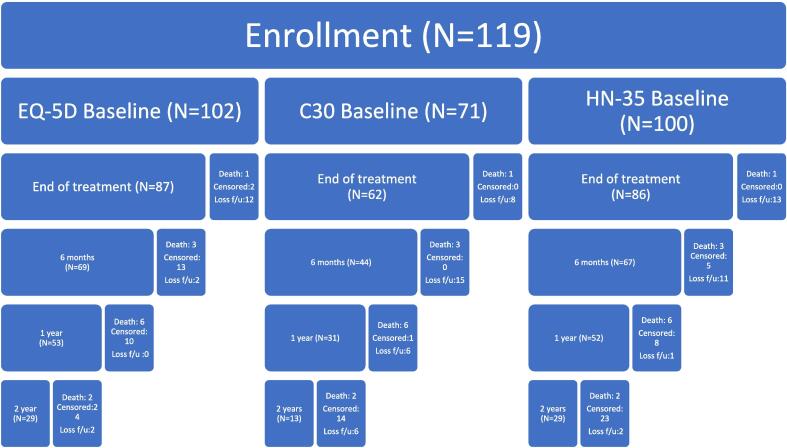
Flow chart of enrollment.

In this study, 119 HNC patients were included (Fig. 1). Patients' characteristics and demographic data before and after multiple imputations are shown in Table 1. The mean age of the study population was 61.5 years old, with 68.6 % male. Almost half (48.8 %) of the included patients had been diagnosed with oropharyngeal cancer. The remaining cases distributed across unknown primary (25.6 %), nasopharynx (9.1 %), oral (9.1 %), hypopharynx (5.0 %) and nasal (2.5 %). The advanced cancer cases (stage III and IV) dominated this study population, with 38 % of stage III and 54.6 % of stage IV. Over sixty per cent of the patients received chemotherapy, while only ten patients underwent surgery before the PT.

### Prevalence of radiation-related toxicities

**Fig. 2:**
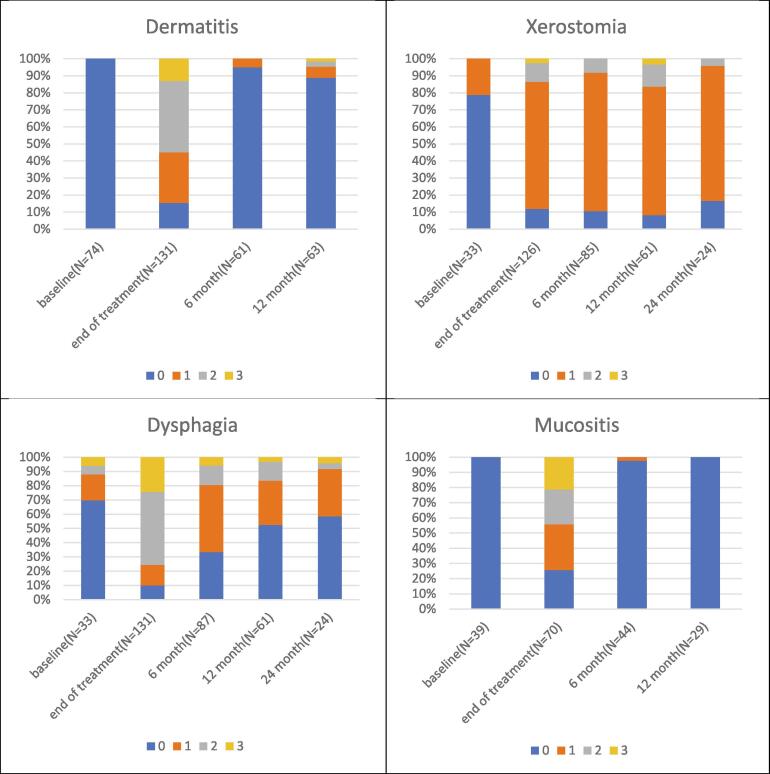
The prevalence of radiation-related toxicities.

The prevalence of radiation-related toxicities observed in this study was demonstrated in Fig. 2. The prevalence of dermatitis, xerostomia, mucositis, and dysphagia peaked at the end of PT and slowly reduced in the following year. However, only dermatitis, mucositis and dysphagia dropped back to baseline levels, while xerostomia remained present at higher levels compared to baseline during the entire observation period.

### Time trend of patients’ function and symptom

**Fig. 3:**
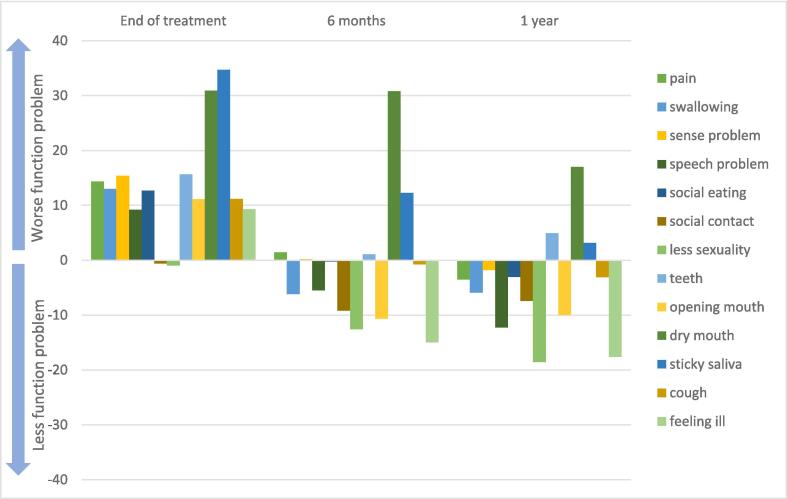
The patients’ functions and symptoms change over time compared to baseline.

A GEE model was employed to estimate the patients' functions and severity of symptom changes over time to further explore the impact of radiation-related toxicities (Model shown in appendix). The results in Fig. 3 show that compared with the patient's baseline conditions, patients reported worse in function of swallowing, sense, speech, and social eating and showed a series of treatment-related symptoms (i.e., dry mouth, sticky saliva, cough and feeling ill) at the end of treatment. At the six months follow-up, most of the functions had been restored, and less severity in the symptoms was reported. Of all functions and symptoms, only pain, problems with teeth, dry mouth and sticky saliva had not recovered to the baseline level. The result of the one-year follow-up indicated that pain had improved within these six months. However, the problem with teeth, dry mouth and sticky saliva symptoms had not disappeared one year after the end of treatment.

### Generic HRQoL analysis: Time trend

**Fig. 4:**
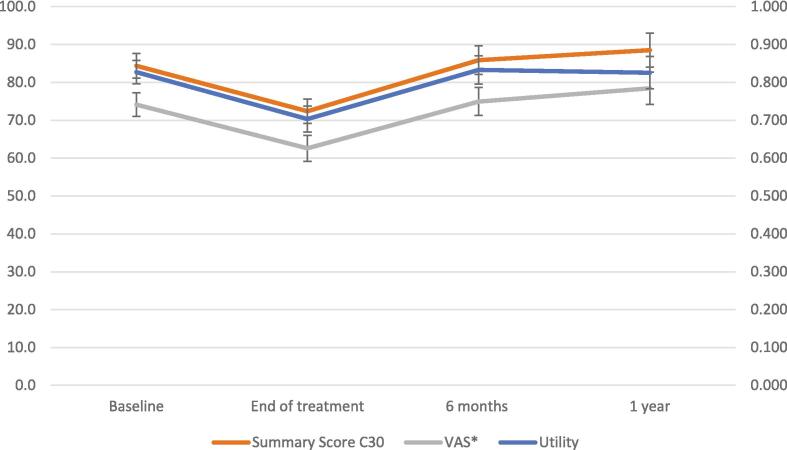
The utility, VAS, and summary score C30 change over time. (The bar represents the 95% CI). *VAS: Visual Analogue Scale.

Regression analysis with the GEE model was performed to estimate the utility and HRQoL (VAS and summary score), and time trends (Model for utility shown in Table 2; models for VAS and summary score shown in appendix). With treatment options (chemotherapy and surgery), cancer characteristic, and disease progression controlled, this estimation aimed to present a general idea of the impact of radiation-related toxicities and how it changed over time. The result revealed that from baseline, the patients' utility score decreased by 16 % (from 0.83 to 0.70) after finishing the PT course (Fig. 4). Six months after the end of the treatment, the patients' utility score returned to 0.83, which was similar to the patient's baseline utility score. The patients' utility remained relatively stable in the following year. Notably, patients with positive HPV status demonstrate significantly better utility compared to those with negative HPV status.

**Table 2:** The generalized estimating equation model for time trends of utility.

**Variable (benchmark)**	**Coefficient**	**SE**	**P**	**[95 % conf.**	**interval]**
**Time point** **(Pre-treatment)**					
End of PT	−0.124	0.021	0.000	−0.166	−0.083
6 months	0.005	0.023	0.824	−0.039	0.049
1 year	−0.002	0.025	0.944	−0.051	0.047
2 years	−0.006	0.033	0.844	−0.070	0.057
**Stage (2)**					
3	−0.018	0.049	0.709	−0.114	0.078
4	−0.060	0.048	0.213	−0.154	0.034
**HPV P16 (0)**					
Positive	0.092	0.032	0.004	0.029	0.154
Not performed	0.028	0.035	0.432	−0.041	0.096
**Tumor site (Oral)**					
Oropharynx	−0.029	0.050	0.556	−0.126	0.068
Hypopharynx	−0.037	0.068	0.588	−0.170	0.096
Nasopharynx	0.026	0.056	0.637	−0.083	0.136
Nasal	−0.127	0.088	0.149	−0.301	0.046
Unknown Primary	−0.033	0.051	0.508	−0.132	0.066
**Disease progression (free of progression)**					
local	0.015	0.072	0.837	−0.127	0.157
Reginal	0.044	0.130	0.735	−0.210	0.298
Metastasis	0.052	0.092	0.571	−0.128	0.232
**Cons**	0.846	0.062	0.000	0.725	0.968

A similar trend was also observed in the change in summary score and visual analog scale (VAS) over time. The patients' summary scores showed further improvement (increased from 84.5 (95 %CI: 81.3–87.7) to 88.7 (95 %CI: 84.2–93.2)) at one-year follow-up. A statistically significant correlation (C30 and utility: r = 0.78; p < 0.0000; C30 and VAS: r = 0.74; p < 0.000; VAS and utility: r = 0.72; p < 0.000) was observed between utility and both HRQoL values (utility and summary score; VAS and summary score; VAS and utility).

Due to the loss of follow-up and limited observational period (later enrolled patients have yet to reach two years follow-ups), the sample size in the later follow-up period is small. This limitation led to higher uncertainty around the utility estimation, as reflected in the 95 % confidence interval range. The complete case analysis at the baseline resulted in an almost identical utility value to the MI analysis (0.83 (95 %CI: 0.80–0.86) vs 0.83 (95 %CI: 0.80–0.86)).

### Disutility of radiation-related toxicity

The GEE model was also used to estimate the impact of radiation-related toxicities on patients' utility. The patients with dysphagia, dermatitis, or xerostomia showed no statistically significant influence on patients’ utility (Table 3). The patients with mucositis present 0.08 point (95 %CI: −0.13 to −0.03) lower in their utility compared with the one without.

**Table 3:** The generalized estimating equation model for utility.

**Variable (benchmark)**	**Coefficient**	**SE**	**P**	**[95 % conf.**	**interval]**
**Chemotherapy** **(No chemotherapy)**					
Concurrent	0.050	0.030	0.118	−0.012	0.104
**Stage (2)**					
3	−0.033	0.051	0.517	−0.134	0.067
4	−0.090	0.052	0.083	−0.192	0.012
**Dermatitis (Grade 0)**					
Grade 1–4	−0.041	0.024	0.089	−0.088	0.006
**Xerostomia (Grade 0)**					
Grade 1–4	0.009	0.024	0.695	−0.037	0.056
**Mucositis (Grade 0)**					
Grade 1–4	−0.084	0.026	0.001	−0.134	−0.034
**Dysphagia (Grade 0)**					
Grade 1–4	−0.047	0.025	0.058	−0.090	0.002
**Disease progression (free of progression)**					
local	0.050	0.073	0.494	−0.093	0.194
Reginal	0.017	0.132	0.896	−0.242	0.277
Metastasis	0.096	0.093	0.304	−0.088	0.280
**Cons**	0.872	0.046	0.000	0.782	0.962

The disutility of each radiation-related toxicity estimated in separate models was shown in Table 4. Compared to patients without any toxicity, patients experienced with toxicity have significantly lower utility and the utility decreased with increasing number of toxicities. With GEE models specific for each radiation-related toxicity, a different estimate of toxicity disutility was obtained. With this approach, patients with mucositis, dysphagia, xerostomia, and dermatitis showed a significant decrease in utility (Models were included in the appendix).

**Table 4:** The disutility of radiation-related toxicity (Models were included in the appendix).

	Disutility	95 % CI	

Dysphagia	−0.071	−0.107	−0.036
Xerostomia	−0.049	−0.084	−0.014
Dermatitis	−0.076	−0.112	−0.040
Mucositis	−0.097	−0.148	−0.046
Any toxicity	−0.047	−0.085	−0.009
One more toxicity	−0.021	−0.030	−0.011

## Discussion

Our study showed that the patients' utility decreased at the end of PT and returned to a baseline score six months afterwards (Fig. 4). The patients' utility scores remained at the similar level as the baseline during the later follow-up periods. The utility change in patients who received PT was also observed in other studies [21]. However, due to different study designs, the HRQoL at the end of treatment was unavailable in these studies. The measuring time points used in the current study accurately reflected the influence of acute treatment-related toxicities.

This study showed a significant correlation between utility and HRQoL trends using either of EQ-5D or QLQ-C30 demonstrating EQ-5D is detecting a similar trend than the disease specific instruments. Prior research established such correlation only in esophageal cancer [22]. This study extends this correlation to the broader HNC population, yielding two notable implications: 1) the potential of using the existing QLQ-C30 as a proxy for utility. Health economists could use the QLQ-C30 results without sacrificing the comparability of HRQoL outcomes across diseases and 2) that economic evaluations of HNC treatments could use utility scores of EQ-5D to measure the benefit of treatments with minimal risk that these values would deviate strongly from disease specific measures.

Our findings also revealed that patients experienced a peak in function loss and symptom burden at the end of the treatment, followed by an improvement six months post-treatment. This validates the observations made in prior studies [23], [24], [25], despite variations in measurement instruments. Two of these studies indicated that symptom burden reached its peak at the end of PT, around six weeks after treatment initiation [23], [25]. However, during the chronic phase, three months post-therapy, symptom burden exhibited a marked decrease. Moreover, an investigation highlighted that xerostomia-related quality of life (XeQoLS) reached its lowest point at the end of treatment, with improvement noted in the tenth week following the treatment [24].

This study is the first to report the disutility of dermatitis and mucositis in HNC patients treated with PT and to further verify the existing disutility of xerostomia and dysphagia with panel data analysis. According to our results, the disutility caused by abovementioned toxicities was not statistically significant which might be related to the small sample size (e.g., 95 %CI ranged from −0.04 to 0.06 for xerostomia). However, significant disutility values were observed in the separated GEE model for each toxicity (Table 4; models shown in the appendix). Only one study proffered an estimation of the impact of toxicities on patients' utility [11]. Within this cross-sectional study, the authors revealed that xerostomia and dysphagia significantly negatively impacted patients' utility (ranging from −0.06 to −0.12). Compared to the present study (Table 3), Ramaekers et al. reported higher absolute disutility values. This variance might stem from divergent study designs and variable inclusions. Our approach employed panel analysis with longitudinal data, minimizing omitted variable impact via baseline utility adjustments for enhanced precision. Previous study using multivariate analysis to estimate quality of life for patients receiving different radiotherapy modalities (3DCRT and IMRT) accounted for patient characteristics, cancer stage, tumor site, and chemotherapy, all of which are included in our analysis [26]. Moreover, our study encompassed a broader range of radiation-related toxicities. Notably, strong correlations between mucositis and dysphagia/xerostomia might cause estimation differences (correlation matrix shown in appendix). High coexistence of these toxicity pairs (dysphagia & mucositis, xerostomia & mucositis) could inflate statistics. This deviation in the estimation of the disutility of dysphagia and xerostomia may arise from capturing the negative influence of unmeasured mucositis. Elevated toxicity correlations lead to multicollinearity, yielding less precision and weaker statistical power. We strongly advise to be cautious in extracting disutility values, especially when correlated toxicities are present. Careful consideration of the independence assumption is pivotal for accurate and reliable results.

This study faces several limitations. Firstly, non-response rates varied across instruments: 14.3 % (17/119) for EQ-5D, 16.0 % (19/119) for HN-35, and 40.3 % (48/119) for QLQ-C30. This may have impacted sample representativeness, yet non-responders' characteristics aligned with responders regarding cancer staging and patient traits. Secondly, the generisability of the prevalence of toxicities to RT could be limited. The patients were selected to be eligible for proton therapy according to their toxicity risk reduction, which could lead to selecting a more fragile population than the one receiving RT. Due to this difference in study population, the prevalence of toxicity could be higher than RT population.

Thirdly, attrition rates, especially in the final follow-up, influenced study power. This stemmed from incomplete questionnaires during visits (dropout), analysis predating the two-year follow-up (censored) and death. The accumulated dropout rates of EQ-5D at one year and two years were 13.4 %, and 15.4 %, respectively. During the 2 years follow-up, 12 (10 %) patients died. This could introduce some bias into the results presented in the current study and could have led to a better patient reported outcome. Despite this inevitable uncertainty, the patients who were lost due to follow-up or lost due to death showed no significant distinctions in characteristics or baseline utility with the remaining patients. Importantly, patient utility and HRQoL remained stable, supported by stable toxicity prevalence and patient-reported symptoms.

Optimism bias stemming from long-standing illness and treatment could not be disregarded from the patients' recovery reports [27]. This bias was introduced because of human coping mechanism. Patients might have adjusted their normal utility to a lower, post-illness level. This new normal would make patients easily satisfied with any improvement, which could be reflected in the survey results. Hence, cautious should be taken while interpreting the “fully recovery” results from patient reported outcomes.

Furthermore, although we included all treated patients that responded, and therefore believe the cohort is representative of real-world clinical practice, number of patients treated at the center is inherently limited. Current existing studies focused on proton therapy in head and neck cancer reported lower patient number (range from 69 to 14 patients) compared to this study [24], [25], [13]. A larger sample size would increase the statistical power and allow for more robust analyses, We present in addition to point estimates, SEs to allowing to account for the uncertainty due to our sample size. BY using our estimates and parameters of uncertainty, our findings can still serve as a valuable cornerstone for future research.

Although a direct comparison between PT and RT was not performed in this study, it offers valuable insights into the effects of PT on HRQoL and utility in HNC patients. Our study accurately captured acute treatment-related toxicities by carefully selecting measurement time points, and we are the first to report on the disutility of dermatitis and mucositis in PT-treated HNC patients. While a head-to-head comparison with IMRT would be valuable, variations in study designs and measurement tools present challenges. Nevertheless, our findings provide valuable insight into the impact of radiation-related toxicity on patients' health-related quality of life. This lays an important foundation for understanding the connection between toxicity incidence rates and patients' HRQoL, which will be crucial for future research, including the ongoing national-level study aimed at directly comparing PT and IMRT.

Future research should focus on larger cohort studies with extended observation periods to capture the long-term effects of radiation-related toxicities on patients' quality of life. However, RT and PT are continuously advancing, longer enrolment periods could introduce heterogeneity to the benchmark for HRQoL as the treatment changes over time. For example, the initial treatment planning could be more conservative initially led to higher doses on the organ at risk. As the experience accumulated, the doses to OAR were later reduced. To mitigate the heterogeneity caused by treatment changes, it is crucial to enroll a large number of patients in a relatively short timeframe. Achieving this would be more challenging for PT unless supported by a (inter)national database.

We show that HPV status has a significant correlation with HRQoL. Possible explanations for this could be the relatively good baseline performance and toxicity profiles of this patient group. However, further research is needed to focus on HPV positive OPC to estimate the influence on patients’ HRQOL. Additionally, measures that facilitate patients' adherence (i.e., phone calls or providing rewards) could be introduced to mitigate the attrition problem.

In conclusion, our study showed a decline in patients' utility post-PT, rebounding to baseline after six months. We established a utility-HRQoL estimate to quantify the impact of toxicities in assessing HNC treatments. Additionally, we reported disutility for dermatitis, mucositis, xerostomia, and dysphagia. Caution is advised when estimating disutility values considering toxicity correlations. Overall, our study provides insight on the burden of toxicities and enriches value assessment of PT and other radiotherapy advances.

Conflict of Interest Statement for All Authors

**YHC, MK and MV** report no CoI.

**HMB** has a secondment at HollandPTC and received grants from The Netherlands Organization for Health Research and Development (ZonMw), Medical Delta, the Dutch Healthcare Institute (ZIN) and the CADTH (Canadian Agency for Drugs and Technologies in Health), and an advisory board fee from Pfizer, payments were made to the institute, outside the submitted work and reports to be part of the scientific advisory board of the Dutch Healthcare Institute.

**CAU** has received grants or contracts from Boehringer Ingelheim, Astellas, Celgene, Sanofi, Janssen-Cilag, Bayer, Amgen, Genzyme, Merck, Gilead, Novartis, AstraZeneca, Roche, NIH, and ASCERTAIN, all payments were made to the institute, outside the submitted work.

**MH** reports that Erasmus MC received research grants from Varian Medical Systems a Siemens Healthineers Company, USA, Accuray, USA, Elekta AB, Stockholm Sweden, RaySearch, Stockholm, Sweden, and Siemens Healthineers; HollandPTC received research grants from Varian Medical Systems a Siemens Healthineers Company; received payments paid to Erasmus MC for presentation and support for attending meeting from Accuray, USA outside the submitted work.

Funding Statement

The research is supported by Medical Delta, scientific HollandPTC Medical Delta program on HTA value proposition.

All data generated and analyzed during this study are included in this published article (and its supplementary information files).

## CRediT authorship contribution statement

**Yi Hsuan Chen:** Conceptualization, Methodology, Formal analysis, Data curation, Visualization, Writing – original draft. **Michiel Kroesen:** Investigation, Resources, Writing – review & editing. **Mischa Hoogeman:** Resources, Writing – review & editing, Supervision. **Matthijs Versteegh:** Writing – review & editing. **Carin Uyl-de Groot:** Writing – review & editing, Supervision. **Hedwig M. Blommestein:** Conceptualization, Methodology, Writing – review & editing, Supervision.
