# Supplementary material for: Treatment-related toxicity, utility and patient-reported outcomes of head and neck cancer patients treated with proton therapy: A longitudinal study
Source: Clin Transl Radiat Oncol. 2025 Jan 9;51:100913. doi: 10.1016/j.ctro.2025.100913 (PMC11787426; doi:10.1016/j.ctro.2025.100913)
Supplement: Supplementary Data 1 [file mmc1.docx]

Models for each QLQ-H&N 35 item

Pain

Swallowing

Senses problems

Speech problems

Trouble with social eating

Trouble with social contact

Less sexuality

Teeth

Opening mouth

Dry mouth

Sticky saliva

Coughing

Felt ill

VAS model

C30 summary score model

Separate GEE model for mucositis

Separate GEE model for dysphagia

Separate GEE model for dermatitis

Separate GEE model for xerostomia

The baseline utility of patient who loss follow-up or death during follow-up

Death vs. complete cases

Loss follow-up vs. complete cases

The GEE model for all disutility

Correlation matrix for radiation-related toxicities and HPV_P16
